# GEAR 2.0 ‐ Emergency Care Practices

**DOI:** 10.1002/alz.091837

**Published:** 2025-01-09

**Authors:** Scott M Dresden

**Affiliations:** ^1^ Northwestern University Feinberg School of Medicine, Chicago, IL USA

## Abstract

Persons living with dementia (PLWD) are twice as likely to use the emergency department (ED) and 1.5 times more likely to have an avoidable ED visit than elders without dementia. PLWD have greater comorbidity, incur higher charges, are admitted to hospitals at higher rates, return to EDs at higher rates, and have higher mortality after an ED visit than patients without dementia. When in the ED, they often struggle with the loud, bright, fast‐paced setting and may not be able to give a complete medical history. Additionally, PLWD have different emergency care needs than other patients in the emergency department that may go unrecognized.

Though routine care in the ED is not well suited for PLWD, many experimental changes to components of ED care have been studied for PLWD including comprehensive geriatric assessment, dedicated ED unit, partnering with care partners, optimal pain assessment, fall prevention, delirium screening, admission to hospital at home, palliative care in the ED, and changes to the ED physical environment. This session will review the literature of current and ED care practices for PLWD dementia detection approaches and discuss top research questions generated by the patients, care partners and members of the trans‐disciplinary GEAR ED Care Practices Work Group.

